# Advances in the Treatment Strategies in Hypertension: Present and Future

**DOI:** 10.3390/jcdd9030072

**Published:** 2022-03-03

**Authors:** Paolo Verdecchia, Claudio Cavallini, Fabio Angeli

**Affiliations:** 1 Fondazione Umbra Cuore e Ipertensione-ONLUS, 06100 Perugia, Italy; 2 Division of Cardiology, Hospital S. Maria della Misericordia, 06100 Perugia, Italy; claudio.cavallini@ospedale.perugia.it; 3 Department of Medicine and Surgery, University of Insubria, 21100 Varese, Italy; fabio.angeli@uninsubria.it; 4 Department of Medicine and Cardiopulmonary Rehabilitation, Istituti Clinici Scientifici Maugeri—IRCCS of Tradate, 21049 Tradate, Italy

**Keywords:** hypertension, antihypertensive therapy, renal denervation, diabetes, heart failure, chronic disease

## Abstract

Hypertension is the most frequent chronic and non-communicable disease all over the world, with about 1.5 billion affected individuals worldwide. Its impact is currently growing, particularly in low-income countries. Even in high-income countries, hypertension remains largely underdiagnosed and undertreated, with consequent low rates of blood pressure (BP) control. Notwithstanding the large number of clinical observational studies and randomized trials over the past four decades, it is sad to note that in the last few years there has been an impressive paucity of innovative studies. Research focused on BP mechanisms and novel antihypertensive drugs is slowing dramatically. The present review discusses some advances in the management of hypertensive patients, and could play a clinical role in the years to come. First, digital/health technology is expected to be increasingly used, although some crucial points remain (development of non-intrusive and clinically validated devices for ambulatory BP measurement, robust storing systems enabling rapid analysis of accrued data, physician-patient interactions, etc.). Second, several areas should be better outlined with regard to BP diagnosis and treatment targets. Third, from a therapeutic standpoint, existing antihypertensive drugs, which are generally effective and well tolerated, should be better used by exploiting available and novel free and fixed combinations. In particular, spironolactone and other mineral-corticoid receptor antagonists should be used more frequently to improve BP control. In particular, some drugs initially developed for conditions different from hypertension including heart failure and diabetes have demonstrated to lower BP significantly and should therefore be considered. Finally, renal artery denervation is another procedure that has proven effective in the management of hypertension.

## 1. Introduction

Because of its high prevalence and important clinical impact, hypertension remains a leading contributor to the risk of cardiovascular disease and death [1,2,3,4]. In 2015, about 1.5 billion adults worldwide had a measured office blood pressure (BP) higher than 140 mmHg systolic or 90 mmHg diastolic [5]. According to a recent study, the number of subjects aged 30–79 years with a prior diagnosis of hypertension doubled from 331 million women and 317 million men in 1990 to 626 million women and 652 million men in 2019, despite a stable age-standardized prevalence worldwide [6]. It has been estimated that a systolic BP ≥140 mmHg explains about 70% of the burden of morbidity and mortality worldwide [7,8,9].

Despite such impressive growth, the proportion of treated hypertensive subjects with normal BP (‘controlled hypertension’) remains very low worldwide. It has been estimated that such a proportion approaches 23% in women and 18% in men [6]. Notably, despite an improvement in diagnosis, treatment, and control of hypertension in most developed and high-income countries, important disparities around the world remain. About two-thirds of patients with hypertension actually live in low-income countries [1,10]. Over the past 20 years, there have been no improvements in hypertension awareness, treatment, and control in several countries in sub-Saharan Africa and Oceania [6,11,12,13].

Thus, a first basic consideration is that, although the prevalence and clinical impact of arterial hypertension is consistently growing worldwide, its control remains disappointing, particularly in low-income countries.

A second consideration is that, despite the huge number of observational studies and randomized controlled trials completed over the past four decades, the last few years have been characterized by an impressive paucity of innovative studies. In a comprehensive review, Dzau noted that research on new antihypertensive drugs and therapeutic targets is slowing dramatically [14]. In addition, there has been no recent attempt to develop clinical applications based on the several genomic polymorphisms associated with hypertension [14]. It should be considered that the time lag between initial discovery and the marketing of a new antihypertensive drug may exceed 10 years, with a consequent final cost greater than two billion US dollars [15,16]. Within this framework, industry is directing most efforts to maximize the utilization of old and effective antihypertensive drugs (e.g., development of new combinations, new dosages, etc.) and to redirect these toward hypertension through the use of BP-lowering drugs, initially developed for different diseases (e.g., gliflozines, drugs for heart failure, etc.) [16].

The current review aims to discuss the main trends and perspectives related to the clinical diagnosis and treatment of hypertension over a foreseeable future. More specifically, our review describes the use of new blood-pressure lowering drugs and device-based approaches to achieve better blood pressure control rates and improve cardiovascular outcomes in patients with hypertension are also reviewed. In other words, we offer clinicians some answers to the following question: “what will the management of hypertensive patients be like in 2030?”

## 2. Digital/Health Technology for Diagnosis and Monitoring

Owing to the refinement of digital/health technology, the marketing of electronic devices for remote BP measurement and transmission is growing. Theoretically, these devices have the potential to improve the diagnosis of hypertension and the achievement of an adequate BP control at the population level. Just to create a parallel with diabetes, Dzau noted in his review that the number of apps for diabetes management was about 1800 in 2016, with an impressive increase in digital diabetes marketing [14]. There is no reason why this growth should not apply to the hypertension field in the near future, although the growth of devices and apps for hypertension seems to be much less explosive than that of the management of diabetes [14].

Unfortunately, not all BP measurement devices on the market have been appropriately validated according to existing guidelines [17,18] and some of those show some limitations and shortcomings [14]. Particular attention is being devoted to cuff-less continuous BP monitoring systems as alternative to current cuff-based systems, although their validity and reliability are still under research [14,19,20,21]. We believe that some steps are critical to make a new system reliable:

1. The system should be easily wearable, cheap, and non-intrusive. Systems included in normal smartwatches would be ideal;
2. The system should be validated for accuracy at independent academic or hospital centers. It should allow continuous or almost-continuous BP detection over prolonged periods of time of months or even years;
3. The system should be connectable to an easy-to-use protected digital repository, with software allowing easy BP retrieval over variable periods of time for calculation of appropriate statistical measures (BP averages, variability, etc.) and attached graphics;
4. The system should be easily accessible to doctors, thereby enabling rapid check and response for patients and the suggestion of changes in drug treatment or other measures;
5. Clinical research should urgently identify BP measures retrievable from the system which are more appropriate for the prediction of organ damage and, hopefully, prognosis. In other words, research should identify which BP measurements obtained by the system are more important for clinical decisions.

It is hoped that the application of artificial intelligence to these databases, which are expected to include many different types of biological data for each patient, may help doctors and patients in identifying better strategies for hypertension control, possibly in combination with strategies promoting a healthier diet, better physical activity, and a more intelligent use of drugs. The growing use of ‘tele-medicine’ during the current COVID pandemic should be extended to the management of hypertension. However, there still a long way to go.

## 3. Definition of Hypertension and Establishment of Treatment Targets

Whereas the European Society of Cardiology and the European Society of Hypertension (ESC/ESH) define hypertension by office BP levels ≥140 mmHg systolic or 90 mmHg diastolic, [22] the American Heart Association (AHA), the American College of Cardiology (ACC) and other scientific societies have endorsed a more ‘aggressive’ definition based on office BP values ≥130 mmHg systolic or 80 mmHg diastolic [23]. In addition, the International Society of Hypertension (ISH) adopted the 140/90 mmHg definition [24].

Of note, the more aggressive diagnostic targets endorsed by the US guidelines [23] do no imply that all subjects with office BP in the range of 130–139/80–89 mmHg require drug treatment. Instead, the AHA/ACC guidelines suggest to apply more appropriate life-style measures (weight control, smoking cessation, low-sodium diet, etc.) for these subjects, and to reserve drug treatment for cases of inefficacy of non-pharmacologic measures.

Notably, all guidelines share the recommendation that drug treatment should be started immediately for:

(a) Patients with office BP ≥ 160/100 mmHg regardless of other considerations [22,23,24];
(b) Patients with BP ≥ 140/90 mmHg in the presence of ischemic heart disease, cerebrovascular disease, or heart failure [22,23,24].

All guidelines suggest that drug treatment should be initiated, regardless of other considerations, in patients with BP persistently ≥ 140/90 mmHg in case of inefficacy of life-style measures [22,23,24].

In the case of a BP between 130/80 and 140/90 mmHg, the AHA/AHA guidelines recommend drug treatment in patients with overt cardiovascular disease (i.e., secondary prevention), as well as in patients without overt cardiovascular disease (i.e., primary prevention) if their 10-year risk of cardiovascular disease is ≥10% according to the ASCVD calculator [23].

Available guidelines provide different recommendations in terms of BP targets and definitions of BP control. The ISH and the ESC/ESH guidelines recommend a uniform BP target (<140/90 mmHg), and individualized targets based on age, tolerability, and comorbidities. Conversely, the AHA/ACC guidelines recommend an identical BP target (<130/80 mmHg) in all patients, regardless of age and comorbidities. The potential advantages and disadvantages of these different approaches have been discussed in detail [25,26,27].

Interestingly, the recent 2021 ESC Guidelines on Cardiovascular Prevention [28] introduce the concept that BP targets lower that 130/80 mmHg are always acceptable when a treatment is well tolerated. Such a statement contrasts with prior ESC/ESH guidelines which state that, for safety reasons, systolic BP should not be targeted below 120 mmHg in people younger than 65 years, or below 130 mmHg in older subjects [22].

In summary, hypertension guidelines seem to be oriented towards individualized BP targets according to the general principle that the lowest well-tolerated BP target should be a reasonable target, with the main goal to prevent the most closely BP-related adverse complication of hypertension, which include stroke and heart failure [29].

## 4. Life-Style Measures

Although frequently not utilized by many patients, life-style measures play a pivotal role in BP control. These measures include weight reduction for overweight or obese subjects, a low sodium diet, smoking cessation, alcohol and caffeine limitations, and regular physical activity [22,23]. We should not neglect of dismiss the importance of these measures in the future management of hypertensive patients.

## 5. Chronotherapy

Many studies conducted at independent centers have demonstrated beyond any reasonable doubt the overwhelming prognostic impact of nighttime BP [30,31,32]. On this basis, it has been thought that using antihypertensive drugs in the evening at bedtime, instead of in the morning, could be preferable to control BP, prevent or regress organ damage, and reduce cardiovascular risk. Indeed, some data from a Spanish research group suggested that evening administration could reduce the incidence of major cardiovascular events associated with hypertension [33,34]. However, these data have been harshly criticized for supposed implausibility [35,36]. Other studies have failed to demonstrate a difference between morning and evening administration of antihypertensive drugs in terms of BP control [37,38]. A large randomized study, the TIME study, is underway to provide a final answer to this question [39].

For the time being, it seems reasonable to advise combining morning and evening administration of antihypertensive drugs in selected patients with severe or resistant hypertension, as well as in those with particularly high nighttime BP. Preference should be given to antihypertensive drugs with a long duration of action, capable of covering the entire 24-h period. For example, when choosing among different diuretics, chlorthalidone appears to be the agent of first choice in patients without severe renal failure [40,41]. In a recent study, patients with renal failure (glomerular filtration rate between 15 and 29 mL/min/1.73 m^2^ of body surface area) and uncontrolled hypertension were randomized to chlorthalidone or placebo, with the randomization stratified by prior use of loop diuretics. After 12 weeks of treatment, average 24-h systolic BP was 10.5 mmHg lower in the chlorthalidone group than in the placebo group (*p* < 0.001) [42].

## 6. More Frequent Use of Mineral-Corticoid Receptor Antagonists

In a double-blind, placebo-controlled, within-patient trial (PATHWAY-2) [43], 335 patients with home systolic BP > 130 mmHg, despite maximal therapy, were randomly assigned to receive, for 12 weeks, spironolactone (25–50 mg), bisoprolol (5–10 mg), doxazosin modified release (4–8 mg), and placebo in addition to their baseline BP drugs [43]. Spironolactone reduced home systolic BP more than placebo (–8.7 mm Hg), doxazosin (−4.03 mmHg), and bisoprolol (−4.48 mmHg) [43]. Thus, spironolactone was the most effective antihypertensive agent, regardless of the distribution of baseline plasma renin, although its BP-lowering effect was predicted by plasma renin activity and the aldosterone-renin ratio [44]. Spironolactone reduced thoracic fluid content, differently from the comparative drugs [44].

In a run-out sub-study of PATWAY-2, amiloride, a distal tubular diuretic that inhibits the epithelial sodium channel sensitive to spironolactone, exerted an antihypertensive effect similar to that of spironolactone and was superior to placebo, doxazosin, and bisoprolol [44]. Notably, amiloride lacks the antiandrogen effect of spironolactone, thereby avoiding gynecomastia.

Eplerenone seems to possess a better safety profile than spironolactone and, thus, it might be an alternative to the latter [45,46]. However, hyperkalemia is an adverse effect of mineral-corticoid receptor antagonists that should be carefully considered in patients treated with these drugs.

Anti-aldosterone drugs are currently recommended in patients with resistant hypertension [22,23,47]. It is reasonable to imagine that these drugs will be used more frequently in the future.

## 7. Endothelin Receptor Antagonists

Endothelin regulates vascular tone and BP, producing a powerful vasoconstrictor effect and contributing to the pathogenesis of hypertension [48,49]. It causes neurohormonal and sympathetic activation, hypertensive end-organ damage, fibrosis, endothelial dysfunction, and increased aldosterone synthesis and secretion [48,49].

Furthermore, endothelin-1 (ET-1, the biologically predominant member of the endothelin peptide family) is an endothelial cell-derived peptide with a wide variety of developmental and physiological functions, which include embryogenesis and nociception [50,51]. More specifically, the endothelin system plays a role in regulating the development of the specific neural crest cell population and its derivatives [51].

Interestingly, aging affects the shift in balance of release and/or activity of endothelium-derived substances, including increased expression, release, and activity of ET-1 [50,52]. The finding that excessive production of ET-1 is present in patients and experimental models of aging [50,52] supports the therapeutic benefits of targeting the endothelin system in elderly hypertensive patients [49]. Finally, the possibility that endothelin receptor antagonists may have a role in the treatment of pre-eclampsia (due to the large increase of endothelin in this condition [53]) is still undetermined.

Based on evidence that endothelin is a very potent endogenous vasoconstrictor [54], some trials have evaluated the antihypertensive efficacy and tolerability of drugs capable to block the endothelin-A and endothelin-B receptors. However, results are quite disappointing and the tolerability of endothelin receptor antagonists remains a concern. Indeed, these drugs may cause some unwanted effects, including fluid retention, flushing, and headache [16], which may limit their use in clinical practice.

Development of darusentan, and endothelin-A blocker, was stopped for safety concerns.

A trial with atrasentan in patients with diabetic nephropathy, was stopped for reasons related to low recruitment, and apparently different from safety.

Aprocinentan, a blocker of both endothelin-A and endothelin-B receptors with a very long pharmacological half-life (about 44 h), proved more effective than placebo and lisinopril [55]. Interestingly, this antihypertensive agent seems to exert additional mechanisms beyond the expected beneficial effects of sustained BP-lowering action (including a decrease in renal vascular resistance and left ventricular hypertrophy) supporting the hypothesis that this new agent could expand our antihypertensive arsenal in resistant hypertension [49,56]. Indeed, aprocitentan in patients with resistant hypertension is currently under investigation in the PRECISION phase III trial (ClinicalTrials identifier: NCT03541174).

## 8. Neprilysin Combined with Renin-Angiotensin System Inhibition

The heart produces different natriuretic peptides which include the atrial natriuretic peptide, the B-type natriuretic peptide and the C-type natriuretic peptide [57]. These peptides induce potent natriuresis and vasodilation by acting on different cellular receptors, ultimately leading to enhanced intracellular production of cyclic guanil-cyclase [58].

Neprilysin, a zinc endopeptidase, inactivates, not only the cardiac natriuretic peptides, but also bradykinin [59], thereby inducing vasodilatation and natriuresis resulting from a more prolonged action by these agents [59]. Neprilysin was not developed as monotherapy for clinical use, but combined with drugs that inhibit the renin-angiotensin-aldosterone system.

Omepatrilat was the first-in-class combination of naprilysin with an angiotensin-converting-enzyme inhibitor, but its development was abandoned because of occurrence of severe angioedema [60]. In contrast, LCZ696, a more recently developed combination of neprilysin with the angiotensin II receptor blocker valsartan in the same molecule, proved effective and well tolerated in heart failure [61,62] and hypertension [63].

It is reasonable to foresee that LCZ696 will be increasingly used in the future not only in heart failure, but also for improving BP control, particularly in patients with resistant hypertension. Various reasons are currently favoring a preferential development of this drug in patients with heart failure, but the stage is set for a growing role of this drug in the treatment of hypertension [58,64].

## 9. Angiotensin II Receptor Agonists

Angiotensin II induces vasoconstriction by stimulating the angiotensin 1 receptors, and vasodilatation by stimulating the angiotensin 2 receptors. In experimental and clinical settings, stimulation of angiotensin 2 receptors inhibits fibrosis [65] and induces vasodilatation, natriuresis, and blood pressure reduction [66,67]. Consequently, angiotensin II receptor agonists display an interesting antihypertensive potential and are currently investigated for efficacy and safety [68,69].

## 10. Sodium-Glucose Cotrasporter-2 Inhibitors

About 97% of glucose secreted at glomerular level is reabsorbed in the proximal renal tubule through the sodium-glucose cotrasporter-2 receptors (SGLT2) [70]. The remaining 3% is reabsorbed by the SGLT1 receptors, also located in the proximal tubule [70]. Inhibition of SGLT2 and SGLT1 receptors results in an increased excretion of glucose with urines with consequent reduction of hemoglobin A1C [70,71].

In pivotal phase III clinical trials, selective SGLT2 receptor inhibitors empagliflozin, canagliflozin, dapagliflozin and ertugliflozin modestly reduced systolic and diastolic BP through various mechanisms which may include natriuresis, osmotic diuresis and reduction of the sympathetic tone [72]. These drugs induced a marked reduction in the risk of heart failure [72]. In patients with heart failure and reduced ejection fraction (HFrEF), both with and without diabetes, empagliflozin and dapagliflozin reduced cardiovascular mortality and the need of re-hospitalizations for heart failure [73,74]. In patients with heart failure with preserved ejection fraction (HFpEF), empagliflozin significantly reduced the risk of cardiovascular death or hospitalization for heart failure by 21% [75].

In the EMPA-REG BP trial, empagliflozin 10 mg and 25 mg reduced 24-h ambulatory BP by 3.44/4.16 mmHg more than placebo and the degree of antihypertensive effect was comparable in the presence of none, one or more than one antihypertensive drug [76].

**Figure 1:**
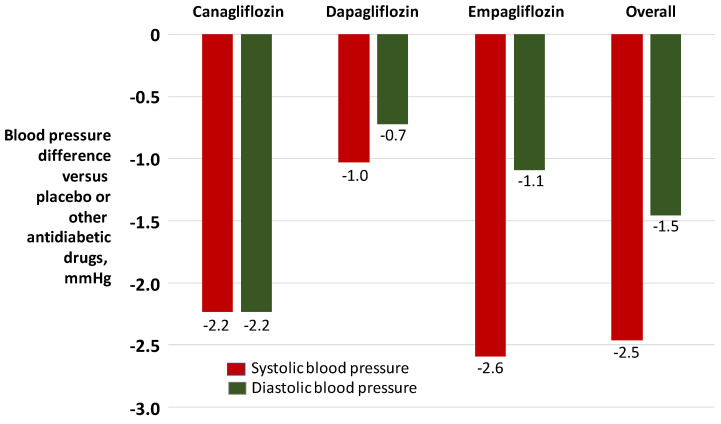
Blood pressure lowering effects of sodium-glucose cotransport-2 inhibitors on blood pressure in patients with diabetes mellitus. Adapted from Mazidi and coworkers [78].

According to available meta-analyses (Figure 1), the degree of BP reduction induced by SGLT2 receptor antagonists appears to be numerically modest [77,78,79]. However, these drugs have the advantage of reducing glomerular hyperfiltration through vasoconstriction of the afferent arterioles, thereby reducing proteinuria and progression of kidney disease, with measurable nefroprotective effects in terms of major renal events [80].

Although these drugs are generally well tolerated, concerns have been raised about volume depletion, acute kidney injury, and genital infections as potential adverse effects. The SGLT2 receptor inhibitors have been recently suggested by guidelines as first-line antidiabetic drugs in patients with diabetes at high or very high cardiovascular risk due to organ damage or concomitant risk factors [72]. In the future, the use of these drugs is expected to be more recommended for hypertensive patients with diabetes or heart failure, although their place in subjects with uncomplicated hypertension is still under evaluation.

## 11. Renal Denervation

Renal sympathetic overactivity contributes to the development and progression of hypertension [81,82,83]. Renal denervation in experimental models of hypertension has been shown to reduce BP and improve renal function, which laid the foundation for its introduction to clinical practice [83,84].

Some clinical trials published over the past 15 years generated many expectations on the clinical utility of renal denervation [85]. Unfortunately, the SIMPLICITY HTN-3 trials failed to demonstrate the superiority of renal denervation over sham control in terms of BP lowering effect [86]. However, the SIMPLICITY HTN-3 trials had several methodological shortcomings. Just to mention some of these limitations, the study erroneously included patients with secondary hypertension (hyperaldosteronism, etc.), 34% of operators had executed only one denervation procedure in the past, drug treatment was much more intense in the ‘sham’ control group than in the denervation group, denervation was not ‘complete’ (not all quadrants of renal artery were ablated) in 75% of cases. Thus, the entire issue was reconsidered, with planning and execution of newer better-designed clinical trials, which provided positive results [87,88,89].

**Figure 2:**
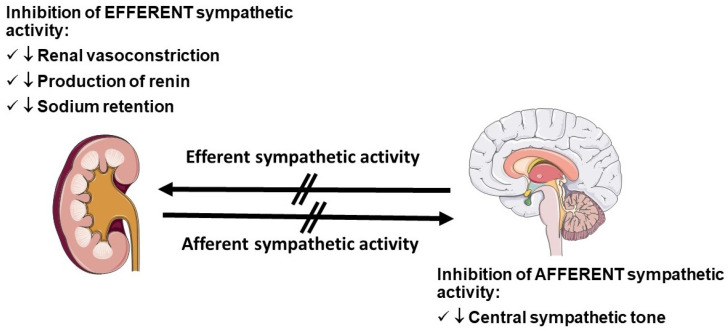
The main effects of inhibition of afferent and efferent sympathetic activity induced by renal denervation.

Renal artery denervation has a strong pathophysiological rationale to justify a significant BP lowering effect (Figure 2).

It is well known that sympathetic firing originating from the ganglia located in the central nervous system induces a variety of effects at cardiac, renal, vascular, and muscular levels that ultimately trigger BP elevation. Several mechanistic studies have demonstrated that ablation of efferent and afferent renal nerves is followed by a reduction of the neural ‘bursts’ of sympathetic activity, detectable by neurography, with parallel reduction in BP [90]. Furthermore, industry produced newer and more effective denervation catheters over the past few years.

**Figure 3:**
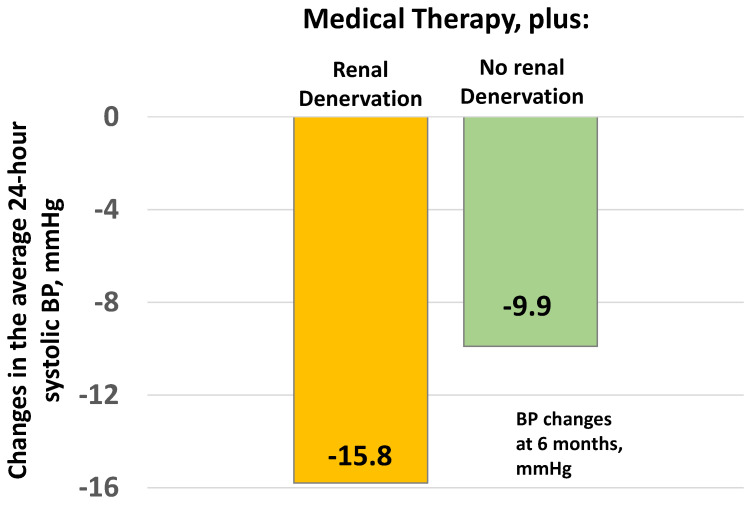
Changes in 24-h systolic BP at 6 months in patients with renal denervation and in a control group not receiving renal denervation. Adapted from Azizi and coworkers [91].

In the DENERHTN trial (Figure 3), 106 patients with resistant hypertension were randomized to continue drug treatment with or without renal denervation using radiofrequency. The ‘no renal denervation’ group did not include a sham procedure. Average 24-h systolic BP at 6 months after the procedure fell by 15.8 mmHg in the denervation group and 9.9 mmHg in the no denervation group (*p* = 0.03) [91].

**Figure 4:**
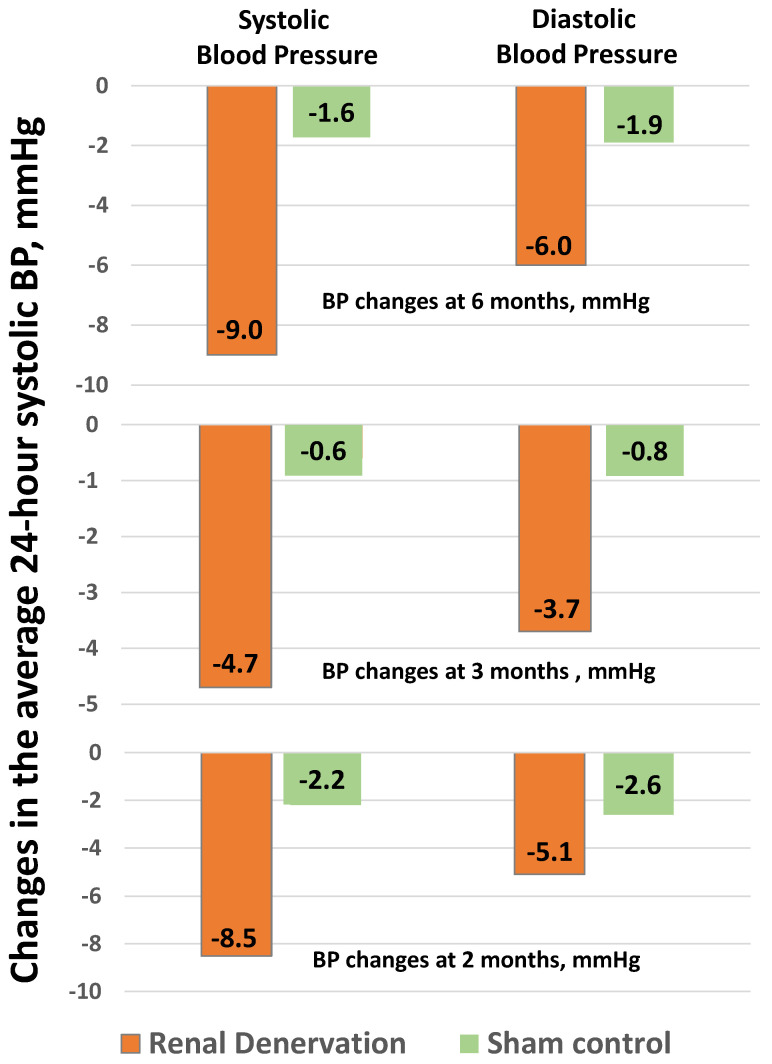
Changes in 24-h systolic BP at different time intervals in patients treated with renal denervation or sham control. Adapted from Azizi and coworkers [87], Bohm and coworkers [88] and Kandzari and coworkers [89].

In the SPYRAL HTN-ON MED trial (Figure 4), 80 patients with resistant hypertension were randomized to continue drug treatment with or without (sham procedure) renal denervation using radiofrequency. Average 24-h systolic BP at 6 months after the procedure fell by 9.0 mmHg with renal denervation and only 1.6 mmHg with the sham procedure (*p* < 0.05) [87]. In the SPYRAL HTN-OFF MED Pivotal trial (Figure 4), 331 untreated patients were randomized to a sham procedure or renal denervation using radiofrequency. Average 24-h systolic BP at 3 months after the procedure fell by 4.7 mmHg after renal denervation and by 0.6 mmHg after the sham procedure (*p* < 0.05) [88]. Finally, in the RADIANCE-HTN SOLO (Figure 4), 331 untreated patients were randomized to a Sham procedure or renal denervation using high frequency ultrasounds. Average 24-h systolic BP at 3 months after the procedure fell by 8.5 mmHg after renal denervation and by 2.2 mmHg after the sham procedure (*p* < 0.05) [89]. Overall, these new trials convincingly demonstrated the superiority of renal denervation over the sham procedure in terms of BP reduction at 3 to 6 months.

**Figure 5:**
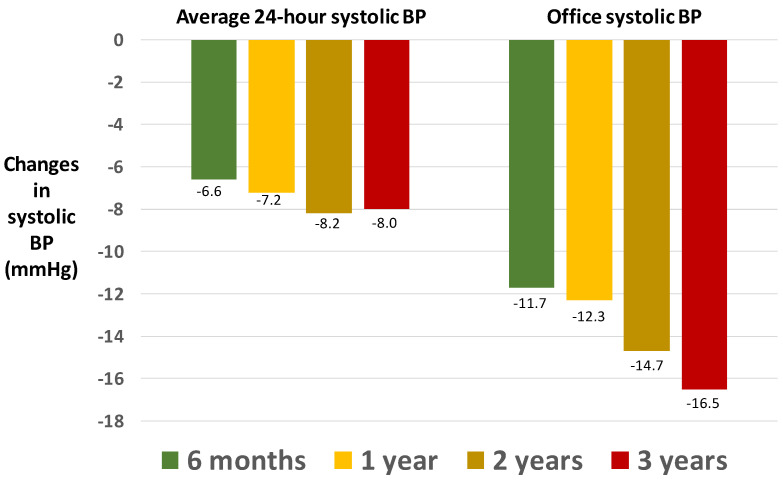
Long-term reduction in systolic BP in the open, non-comparative Global SIMPLICITY Registry. Adapted from Mahfoud and coworkers [89,92].

Concerns remain about the persistence of the antihypertensive effect over the long term. However, encouraging results came from the open and not comparative Global SIMPLICITY Registry (Figure 5), which found no attenuation, or even a slight potentiation, in the antihypertensive effect of renal denervation in the long term (up to three years after the procedure) as compared with pre-procedural levels [92].

A clinical trial compared different techniques of denervation and concluded that the ultrasound technique targeted on both main renal artery and its bifurcations was superior to the radiofrequency technique targeted on the main renal artery alone [93].

In conclusion, renal artery denervation has the potential to be furtherly adopted in clinical practice over the next few years. The main contraindication remains renal artery stenosis, which is rare in unselected patients, but relatively higher, up to 30%, in those with more severe or resistant hypertension [94]. Procedural complications of renal denervation (renal artery dissection, post-procedural stenosis) are extremely rare [95].

Ongoing studies should lead to identification of patients more likely to benefit from renal denervation in terms of BP lowering effect. According to a position paper of the Italian Society of Hypertension [95], some clinical conditions (Table 1) should dictate a preferential indication to renal denervation.

Of note, patients with moderate to severe chronic kidney disease were excluded from large international trials, and smaller studies suggest limited utility in this population [96].

Despite the evidence that renal denervation is associated with a low incidence, of mostly, minor complications [94,95,97], an aspect to consider is the question of renal artery stenosis after this procedure. Some anecdotal reports of renal artery stenosis after renal denervation were published, occurring 5–6 months after a successful procedure and leading to a re-elevation of previously depressed BP [98,99,100,101,102,103].

Thus, when considering renal nerve ablation, arteries with visible stenosis, with calcification or atheromatous plaques, represent relative contraindications [77,78,80].

Finally, available data argue in favor of an incomplete and insufficient ablation of renal sympathetic nerves as a major cause of inadequate BP responses to catheter-based interventions. Indeed, it is not entirely clear whether catheter design and energy delivery may influence the variability of the response to renal nerve ablation and the risk of the development of renal artery stenosis [94,104].

## 12. Conclusions

BP is a very potent risk factor. Unfortunately, at variance with other risk factors, such as serum cholesterol, glucose or creatinine, or even body weight or cigarette smoking, BP is extremely variable over time and this may leave uncertainty or even frustration on the real value of what we are measuring. BP recording remains generally intrusive and the precise rules for a correct BP measurement in the clinical practice are scarcely known. Many patients still do not realize that is perfectly normal to find out BP values of 125/70 and 145/85 mmHg at distance of few minutes. Clearly, such imprecision in diagnosis does not help to achieve BP control when needed.

It is hoped that the future will lead to development of accurate and non-intrusive devices for BP measurement in the long-term. From a therapeutic standpoint, we currently dispose of many effective and well tolerated antihypertensive drugs, but a long way is still to do for an optimal use of these drugs, alone or in combination. Unfortunately, research on new antihypertensive drugs dramatically slowed over the past few years. We agree with Bhudia that the future in the management of hypertensive patients remains uncertain [105]. However, significant progress is likely to come over the next few years from a combination of education and technology worldwide.

## Figures and Tables

**Table 1:** Clinical features of patients who may be candidates to renal denervation. Adapted from a position paper of the Italian Society of Hypertension [95].

**(A)** **Hypertension not controlled by combinations of renin-angiotensin-aldosterone system blockers, diuretics and calcium channel blockers at maximal tolerated dose** Adverse reactions with spironolattone Low adherence to treatment Systo-diastolic hypertension Vascular damage not diffused High or very high cardiovascular risk Patient preference **(B)** **Essential hypertension stage 1 or 2, either untreated or not controlled with 1–2 drugs** Adverse reaction to several antihypertensive drugs Low adherence to treatment High or very high cardiovascular risk Atrial fibrillation with planned ablation Patient preference
